# MicroRNA-216b targets *HK2* to potentiate autophagy and apoptosis of breast cancer cells *via* the mTOR signaling pathway

**DOI:** 10.7150/ijbs.48933

**Published:** 2021-07-13

**Authors:** Ting Liu, Ping Ye, Yuanyuan Ye, Baosan Han

**Affiliations:** 1 The Affiliated Hospital of Qingdao University, Qingdao 266000, P.R. China.; 2 Department of General Surgery, Xinhua Hospital, School of Medicine, Shanghai Jiao Tong University, Shanghai 200092, P.R. China; 3 School of Medical Instrument and Food Engineering, University of Shanghai for Science and Technology, Shanghai 200093, P.R. China

**Keywords:** MicroRNA-216b, HK2, mTOR signaling pathway, Breast cancer, Autophagy

## Abstract

Patients suffering from breast cancer (BC) still have a poor response to treatments, even though early detection and improved therapy have contributed to a reduced mortality. Recent studies have been inspired on the association between microRNAs (miRs) and therapies of BC. The current study set out to investigate the role of miR-216b in BC, and further analyze the underlining mechanism. Firstly, hexokinase 2 (HK2) and miR-216b were characterized in BC tissues and cells by RT-qPCR and Western blot assay. In addition, the interaction between HK2 and miR-216b was analyzed using dual luciferase reporter assay. BC cells were further transfected with a series of miR-216b mimic or inhibitor, or siRNA targeting *HK2*, so as to analyze the regulatory mechanism of miR-216b, *HK2* and mammalian target of rapamycin (mTOR) signaling pathway, and to further explore their regulation in BC cellular behaviors. The results demonstrated that HK2 was highly expressed and miR-216b was poorly expressed in BC cells and tissues. HK2 was also verified as a target of miR-216b with online databases and dual luciferase reporter assay. Functionally, miR-216b was found to be closely associated with BC progression *via* inactivating mTOR signaling pathway by targeting *HK2*. Moreover, cell viability, migration and invasion were reduced as a result of miR-216b upregulation or *HK2* silencing, while autophagy, cell cycle arrest and apoptosis were induced. Taken together, our findings indicated that miR-216b down-regulates *HK2* to inactivate the mTOR signaling pathway, thus inhibiting the progression of BC. Hence, this study highlighted a novel target for BC treatment.

## Introduction

Breast cancer (BC) is a heterogeneous disease of varying subtypes, and each subtype presents with a different overall survival 1, 2. Over the years, age, height, reproductive factors (e.g., nulliparity, older age at first birth), use of exogenous hormones, and family history have been elucidated as risk factors for BC. In addition, lifestyle factors such as alcohol consumption, lack of physical activity, and postmenopausal obesity have also been attributed to occurrence of BC to some degree 3. Hence, early detection is tantamount in controlling the disease mortality all over the world 4. Accurate estimation for the prognosis of each case would both offer beneficial clinical decision-making and individualized design of follow-up management after surgical treatment 5. Moreover, some patients still exhibit poor responses to treatment, even though early detection and improved therapy have contributed to reduced mortality 6. Fortunately, the role of microRNAs (miRs) on the development of BC has been investigated in recent years 7, and has been highlighted to offer promising therapeutic targets for patients plagued by BC.

One such miR, miR-216b is known to be extensively implicated in a multitude of cellular processes, including proliferation, invasion, and epithelial-mesenchymal-transition (EMT) 8; and miR-216b has also been identified to function as a tumor suppressor in various human cancers 9, 10. In addition, miR-216b also confers a tumor suppressive role in BC 11. A prior study has also shown that miR-216b can inhibit the proliferation of cervical cancer cells by targeting FOXM1, and miR-216b can further down-regulate FOXM1 downstream targets or its key regulators pRb, c-myc and cyclinD1 12. On the other hand, the hexokinase 2 gene (HK2) has also been associated with cell migration in BC 13. HK2, a regulator of glucose metabolism, is essential for tumor initiation and maintenance 14. More notably, miR-216b is known to directly-target the 3'-UTR of HK2 to suppress uveal melanoma tumor growth 15. However, the relationship between miR-216b and HK2 in BC remains to be defined.

The mammalian target of rapamycin (mTOR) signaling pathway is widely-involved in cellular processes, such as cell growth, proliferation, cellular metabolism, protein synthesis, gene transcription, as well as cell death including apoptosis, autophagy, and necroptosis 16. Moreover, published literature has also shown that the Akt/mTOR signaling pathway contributes to the inhibitory effects of miR-147 on cell proliferation, invasion and migration in BC 17. Strikingly, miR-216b was previously demonstrated to influence the development of endometrial cancer through the mTOR signaling pathway 18. These findings provided a potential network of miR-216b, *HK2* and mTOR signaling pathway in BC. Hence, the current study set out to test the hypothesis that miR-216b may promote autophagy and apoptosis of BC cells *via* regulation of the mTOR signaling pathway and by targeting *HK2*.

## Results

### The potential functional significance of miR-216b and HK2 in BC progression

**Figure 1:**
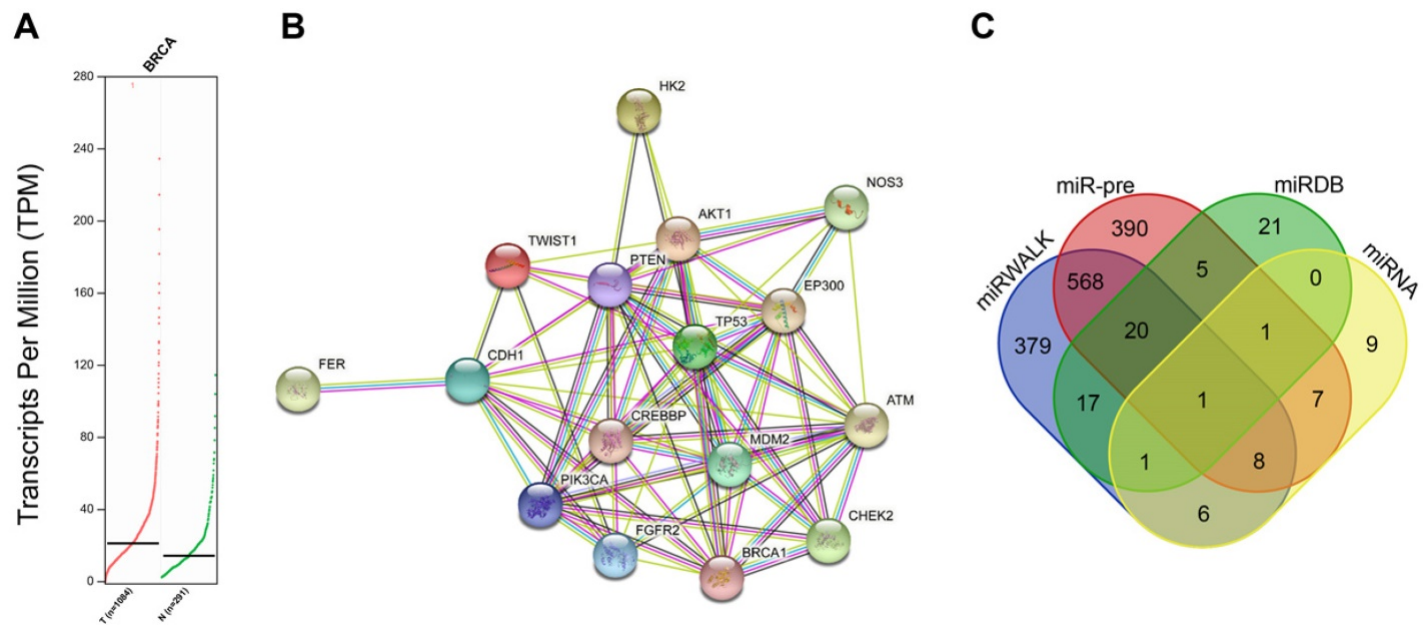
Screening of BC-related genes, signaling pathways and miRs. A, GEPIA2 database reveals expression of *HK2* in BC tissues and normal tissues. B, the interactions between *HK2* and known disease genes analyzed on STRING database; the thickness of line between two genes means the reliability of interaction between two genes, the thicker line indicates a higher reliability; C, the miRs targeting *HK2* predicted by miRWalk and Computational Medicine Center using miR-pre, miRDB Database and microRNA.org.

Firstly, through the GEPIA2 database, it was revealed that *HK2* was highly expressed in BC tissues compared with normal tissues (Fig. 1A). Next, BC-related disease genes were searched and the top 10 genes were selected (Supplementary Table 1). The analysis of PPIs between *HK2* and the known disease genes (Fig. 1B; Supplementary Table 2) showed that *HK2* exhibited close interactions with disease genes (AKT1 and PTEN). Moreover, *HK2* was recently found to be closely related to tumor progression 19. Amparo *et al.* reported that *HK2* is up-regulated in malignant glioma, and cell proliferation in malignant glioma relies on glycolysis 20. In addition, the involvement of *HK2* has also been documented in esophageal squamous cancer, pancreatic cancer and liver cancer 21-23. Also, *HK2*, as a key gene in glycometabolism, has been previously highlighted as a promising therapeutic target for tumors 24, 25. All above data indicated that *HK2* might play a key role in tumor progression. However, mechanism involving the roles *HK2* in BC remains unclear. On the other hand, AKT1 and PTEN are well-known key factors of the PI3K-AKT-mTOR signaling pathway, which is closely related to BC according to the KEGG database (map05224). Moreover, studies have elucidated that cell proliferation, migration and invasion can all be modulated by the PI3K-AKT-mTOR signaling pathway 26-28. Additionally, activated PI3K-AKT-mTOR signaling pathway has been found to promote epithelial-mesenchymal transition (EMT) and angiogenesis in triple-negative BC 29.

To further explore the upstream regulator of *HK2* in BC, we predicted the miRs that could possibly regulate the *HK2* gene. Results from the miRWalk database indicated a total of 2089 potential miRs that could regulate *HK2*. In addition, miR pre-computed tool provided by the Computational Medicine Center (RNA22 tool) predicted another such 2039 miRs. The top 1000 miRs highly-related to *HK2* were selected from each database. Additionally, 66 miRs were also predicted from the miRDB Database and 33 miRs from microRNA.org. Intersections of the above prediction results showed that has-miR-216b was found in all 4 databases (Fig. 1C). Besides, studies have illustrated that has-miR-216b inhibits tumor growth in colorectal cancer, non-small cell lung cancer and cervical cancer 12, 30, 31. Consequently, miR-216b was suggested to probably suppress BC progression *via* inhibition of *HK2* in terms of all above analyses and documentation.

### HK2 is highly expressed in BC

**Figure 2:**
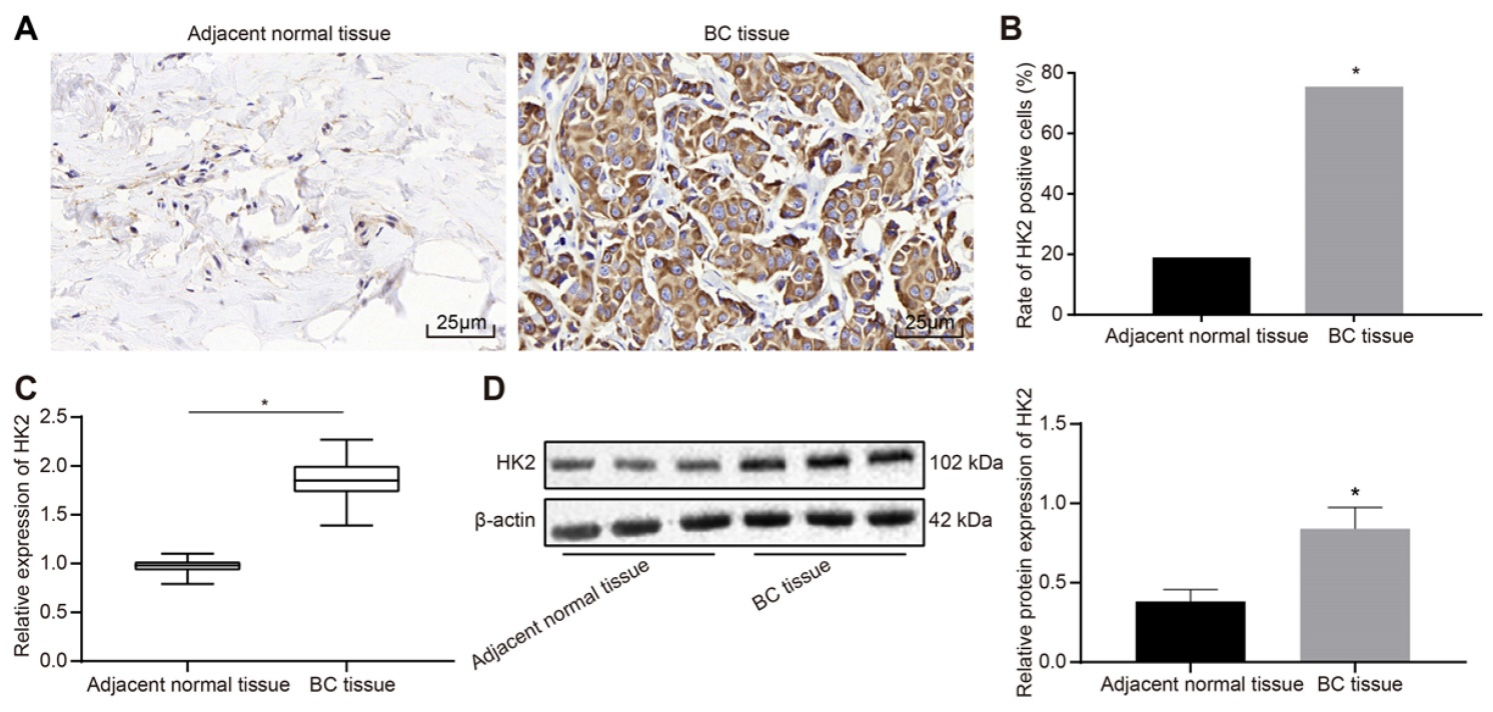
*HK2* is highly expressed in BC tissues. A, immunochemical staining for detection of HK2 expression in BC and adjacent normal tissues (× 400); B, positive rate of HK2; n = 138, the data were analyzed using chi-square test. C, HK2 mRNA expression in BC and adjacent normal tissues determined by RT-qPCR; D, HK2 protein expression in BC and adjacent normal tissues measured by Western blot assay and related statistical data. *, *p* < 0.05 *vs.* the adjacent normal tissues.

The protein expression patterns of HK2 in BC and adjacent normal tissues were detected using immunohistochemistry assay. The results showed that HK2 was highly expressed in BC tissues with the cytoplasm stained with tan coloration, while HK2 was poorly expressed in adjacent normal tissues (Fig. 2A). The positive rate of HK2 protein was calculated to be 73.91% (102/138) in BC tissues, which was significantly higher than 29.71% (41/138) of the adjacent normal tissues (*p* < 0.05) (Fig. 2B). RT-qPCR and Western blot analysis indicated that the mRNA and protein expression of HK2 was elevated in BC tissues relative to adjacent normal tissues (*p* < 0.05) (Fig. 2C-D).

### MiR-216b binds to HK2 3'UTR

**Figure 3:**
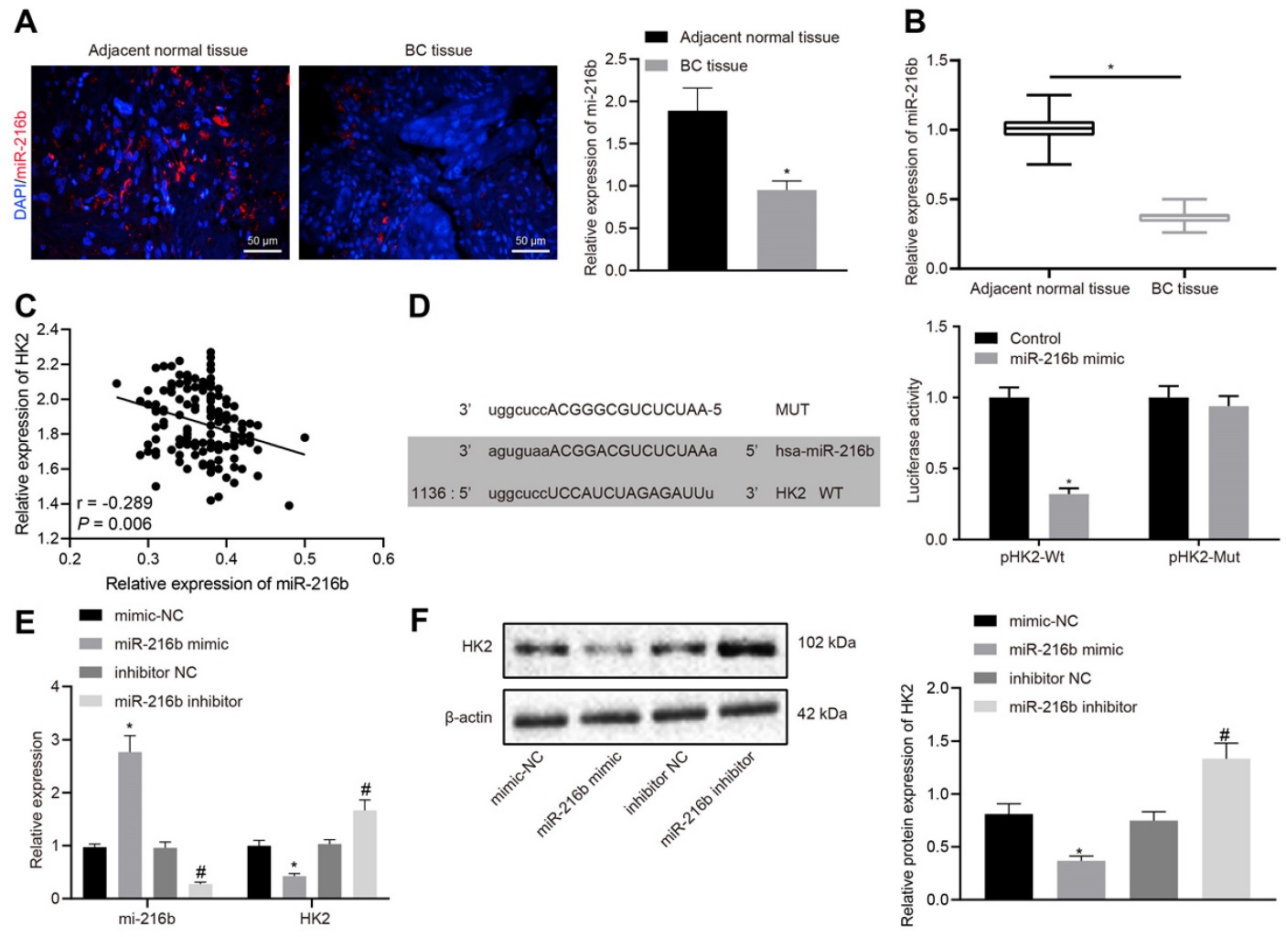
*HK2* is identified as a target gene of miR-216b. A, miR-216b expression determined by FISH (× 200); B, miR-216b expression in BC and adjacent normal tissues determined by RT-qPCR; C, correlation analysis of miR-216b and *HK2* expression in BC tissues. D, the binding site of miR-216b to the *HK2* 3'UTR from the target prediction program microRNA (left); the luciferase activity after treatment by a combination of miR-216b mimics and *HK2*-3'UTR-wt (right); E, miR-216b and HK2 expression after miR-216b expression was altered detected by RT-qPCR; F, HK2 expression after miR-216b expression was altered detected by Western blotting; the experiment was repeated for three times. *, *p* < 0.05 *vs. vs.* Normal tissues, the control group, mimic-NC or inhibitor NC groups.

RT-qPCR and RNA-fluorescence in situ hybridization (FISH) results indicated that miR-216b expression was significantly lower in BC tissues than in the adjacent normal tissues (*p* < 0.05) (Fig. 3A-B), which were negatively-correlated with the expression of *HK2* (Fig. 3C). Furthermore, a target prediction database (microRNA.org) further predicted the presence of a specific binding region between *HK2* 3'UTR and the miR-216b sequence. Subsequent dual luciferase reporter assay results (Fig. 3D) showed that the luciferase activity of p*HK2*-Wt was significantly inhibited by miR-216b mimic compared with the control group (*p* < 0.05). After transfect with miR-216b mimic or inhibitor, miR-216b and HK2 levels were detected using RT-qPCR and Western blotting. The results showed that compared with mimic-NC, miR-216b mimic significantly inhibited the HK2 expression, while miR-216b inhibition brought about the opposite effects on HK2 expression (*p* < 0.05) (Fig. 3E-F). The aforementioned results indicated that miR-216b could target *HK2* and regulate its expression.

### Low expression of miR-216b and high expression of HK2 are associated with BC progression

**Figure 4:**
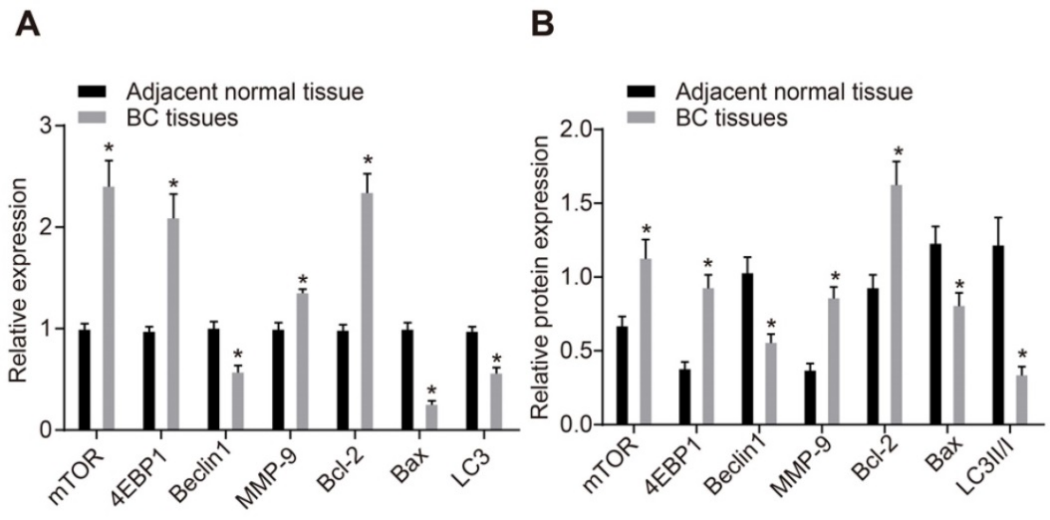
mTOR signaling pathway is activated in BC. A, mRNA expression of mTOR, Bax, Bcl-2, LC3, Beclin1, 4EBP1 and MMP-9, as determined by RT-qPCR; B, Western blot analysis of the protein expression of mTOR, Bax, Bcl-2, LC3, Beclin1, 4EBP1 and MMP-9; n = 138, the data were analyzed using paired *t* test. *, *p* < 0.05 compared with adjacent normal tissues.

As shown in Supplementary Table 3, miR-216b expression and *HK2* mRNA expression exhibited no association with age and histological type. Meanwhile, the miR-216b expression was found to be significantly lower and *HK2* expression was higher in patients with a lesion size > 2 cm, lymph node metastasis (LNM) and at III stage than in those with lesion size ≤ 2 cm, without LNM and at I/II stage (*p* < 0.05). These findings illustrated that low miR-216b expression and high *HK2* expression was associated with BC progression. RT-qPCR and Western blot analysis were then performed to determine the expression patterns of apoptotic factors (Bax and Bcl-2), autophagy-related genes (Beclin1, LC3 and MMP-9), and mTOR signaling pathway-related genes (mTOR and 4EBP1). The results showed that mRNA and protein expression of Beclin1, Bax and LC3 was significantly lower, while that of mTOR, Bcl-2, 4EBP1 and MMP-9 was higher in BC tissues compared to that in adjacent normal tissues (*p* < 0.05) (Fig. 4A, B), suggesting decreased cell apoptosis and autophagy and activated mTOR signaling pathway in BC tissues.

### MiR-216b down-regulates HK2 to block the mTOR signaling pathway

**Figure 5:**
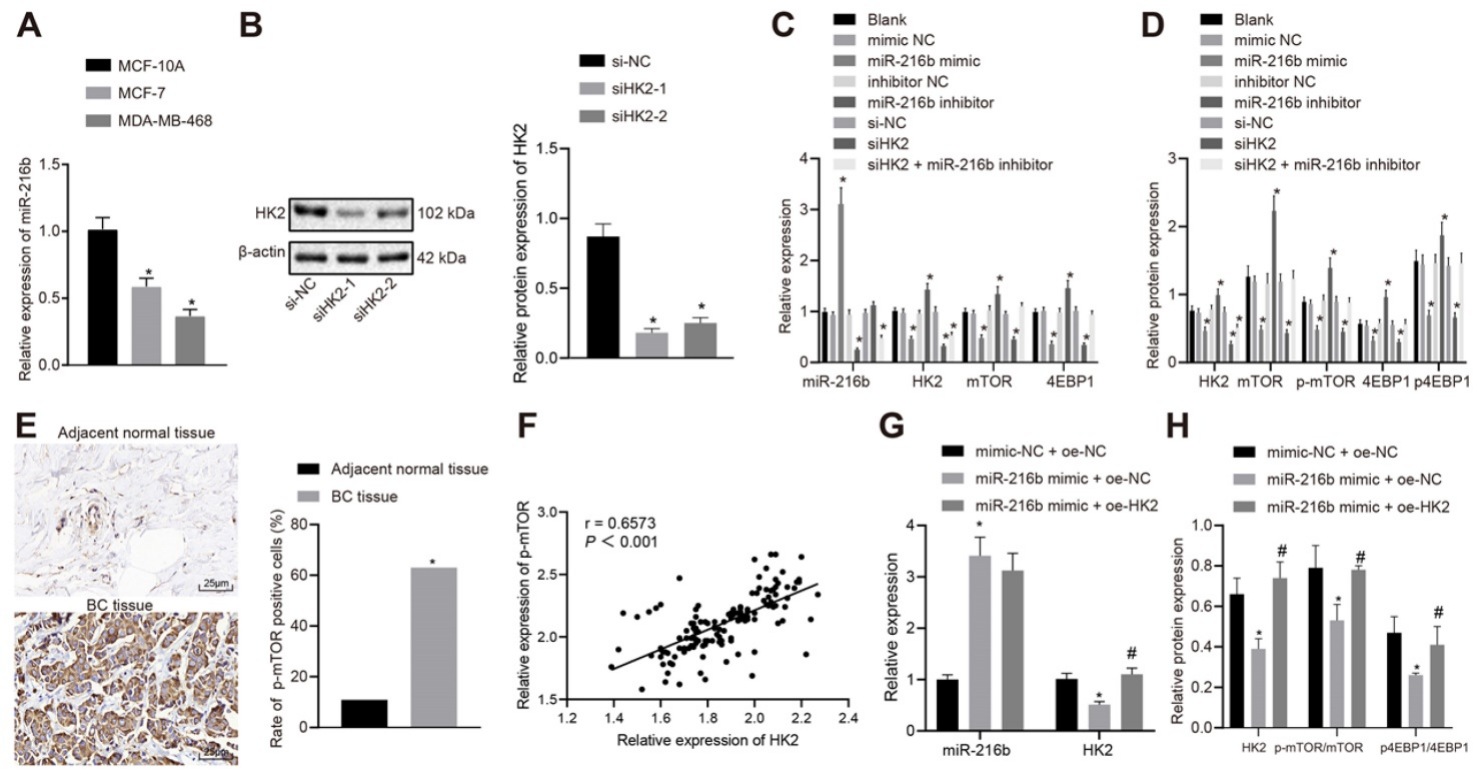
MiR-216b targets *HK2* to block the mTOR signaling pathway. A, miR-216b expression among three tested BC cell lines; B, silencing efficiency of si*HK2*-1 and si*HK2*-2. C, RT-qPCR assay for determination of miR-216b and mRNA expression of *HK2*, mTOR and 4EBP1; D, the protein expression of HK2, mTOR and 4EBP1, along with the extent of mTOR and 4EBP1 phosphorylation measured by Western blot analysis; E, p-mTOR expression in tissues detected by IHC (× 400); F, correlation analysis of p-mTOR and HK2; G, miR-216b and HK2 expression detected by RT-qPCR; H, the protein expression of HK2, mTOR and 4EBP1, along with the extent of mTOR and 4EBP1 phosphorylation measured by Western blot analysis; the experiment was repeated for three times; data were analyzed using one-way ANOVA with Tukey's post hoc test. *, *p* < 0.05 compared with the cells transfected with mimic NC + oe-NC; #, *p* < 0.05 compared with miR-216b mimic +oe-NC.

For cell line selection, miR-216b expression patterns were determined in BC cell lines (MCF-7, MDA-MB-468) and normal human mammary epithelial cell line (MCF-10A) using RT-qPCR. The results showed that, compared with that of MCF-10A cell line, miR-216b expression was decreased in MCF-7 cell line, and further reduced in the MDA-MB-468 cell line (*p* < 0.05) (Fig. 5A); therefore, MDA-MB-468 was used for subsequent experimentation.

As demonstrated by the results from Western blot analysis, si*HK2*-1 was selected for *HK2* knock-down experiments due to more remarkable reduction induced by transfection with si*HK2*-1 (Fig. 5B). There were no significant differences in the expression of mRNA and protein of miR-216b and HK2, mTOR and 4EBP1 between the blank group and NC groups, with no significant differences in the expression of p-mTOR and p4EBP1 (*p* > 0.05). Compared with the NC group, the expression of miR-216b in the miR-216b mimic group was found to be increased significantly (*p* < 0.05), while that of HK2, mTOR and 4EBP1 was decreased, and the expression of p-mTOR and p4EBP1 was also reduced (*p* < 0.05). Meanwhile, the expression of miR-216b in the miR-216b inhibitor group was decreased (*p* < 0.05), while HK2, mTOR and 4EBP1 were up-regulated, and the expression of p-mTOR and p4EBP1 was elevated (*p* < 0.05). Moreover, HK2, mTOR and 4EBP1 expression in si-HK2 group was decreased, and p-mTOR and p4EBP1 expression was also reduced significantly (*p* < 0.05), while that of miR-216b was not significantly altered (*p* > 0.05). After treatment with si-HK2 + miR-216b inhibitor, the expression of miR-216b and HK2 was decreased (*p* < 0.05), while that of mTOR, 4EBP1 and p-mTOR, p4EBP1 did not change significantly (*p* > 0.05) (Fig. 5C-D).

Previous results showed that HK2 was highly expressed in cancer tissues, and the expression of p-mTOR was decreased in BC cells when HK2 was silenced. As a result, we hypothesized that HK2 was positively correlated with p-mTOR, and then detected the expression patterns of p-mTOR in BC tissues and adjacent normal tissues using immunohistochemistry. The results showed that p-mTOR was highly expressed in cancer tissues and positively correlated with HK2 (Fig. 5E-F).

To validate the involvement of the miR-216b-HK2-mTOR regulatory axis in breast cancer cells, we performed a rescue experiment. In MCF-7 cells, RT-qPCR was performed to detect the expression patterns of miR-216b and HK2, and Western blot was applied to detect the expression patterns of mTOR, p-mTOR, 4EBP1 and p4EBP1 in HK2 and mTOR pathways. The results showed that compared with mimic-NC + oe-NC treatment, miR-216b was upregulated but the expression of HK2, p-mTOR and p4EBP1 was inhibited in the miR-216b mimic + oe-NC group Inhibition of HK2, p-mTOR and p4EBP1 expression by miR-216b mimic + oe-NC could be restored by miR-216b mimic + oe-HK2 (*p* < 0.05) (Fig. 5G-H).

### MiR-216b upregulates Beclin1, Bax and LC3 but down-regulates Bcl-2 and MMP-9

**Figure 6:**
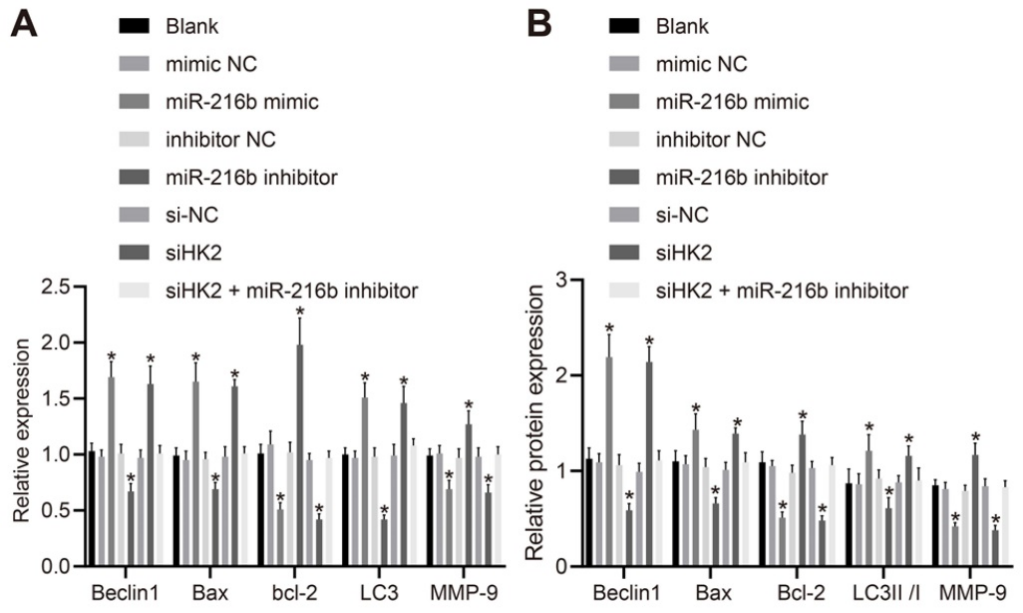
MiR-216b regulates expression of Beclin1, Bax, Bcl-2, MMP-9, LC3, LC3 I and LC3 II. A, mRNA expression of Beclin1, LC3, Bax, Bcl-2 and MMP-9 determined by RT-qPCR; B, the protein expression of Beclin1, Bax, Bcl-2, LC3 I, LC3 II and MMP-9 measured by Western blot analysis; the experiment was repeated for three times; data were analyzed using one-way ANOVA. *,* p* < 0.05 compared with the cells transfected with mimic NC, inhibitor NC or siNC.

To further investigate whether miR-216b could affect BC progression, the cellular expression patterns of Beclin1, Bax, Bcl-2, LC3 and MMP-9 were determined following activation or depletion of miR-216b, or silencing of *HK2*. As depicted in Fig. 6A, B, the mRNA expression of Beclin1, Bax and LC3, protein expression of Beclin1 and Bax and ratio of LC3 II/I were significantly elevated by miR-216b mimic or si*HK2* transfection, while that of Bcl-2 and MMP-9 was markedly down-regulated (*p* < 0.05); and these trends could be reversed following treatment with miR-216b inhibitor (*p* < 0.05). Meanwhile, the co-transfection of si*HK2* and miR-216b inhibitor brought about no significant changes in relation to the aforementioned expression levels (*p* > 0.05). These findings indicated that miR-216b upregulated Beclin1, Bax and LC3 and downregulated Bcl-2 and MMP-9.

### MiR-216b inhibits BC cell proliferation, migration, invasion by targeting HK2

**Figure 7:**
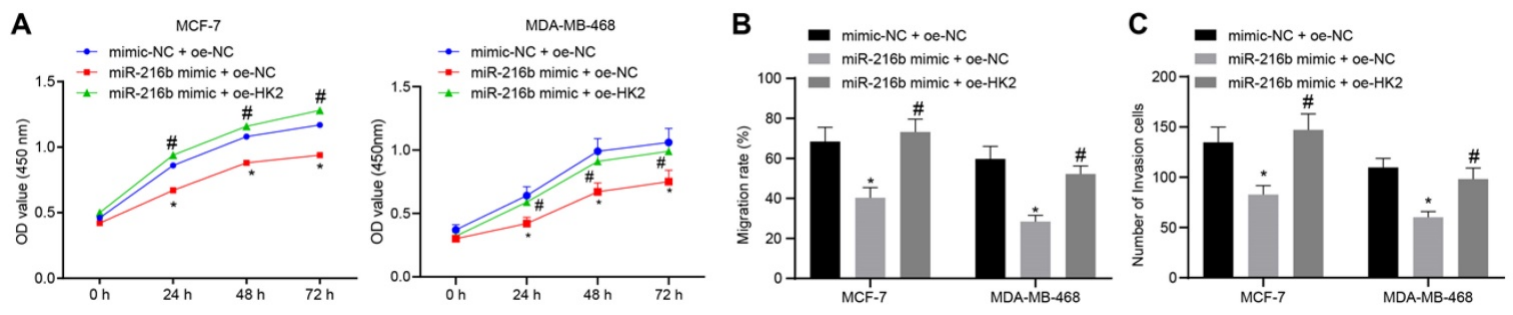
miR-216b inhibits BC cell proliferation, migration and invasion by targeting *HK2*. A, BC cell proliferation in response to miR-216b upregulation or *HK2* overexpression assessed by CCK-8; B, BC cell migration detected by scratch test; C, BC cell invasion detected by Transwell assay; the experiment was repeated for three times; data were analyzed using one-way ANOVA. *, *p* < 0.05 compared with the cells transfected with mimic NC + oe-NC; #, *p* < 0.05 compared with miR-216b mimic +oe-NC.

Next, CCK-8 assay was applied to detect the cell proliferation at the 0 h, 24^th^ h, 48^th^ h and 72^nd^ h time points after transfection. Compared with mimic-NC + oe-NC, cell proliferation was found to be inhibited in the miR-216b mimic + oe-NC group. Meanwhile, miR-216b mimic + oe-HK2 could restore the inhibition of cell proliferation by miR-216b mimic + oe-NC (*p* < 0.05) (Fig. 7A). In addition, scratch assay and Transwell assay were performed to assess the possible regulatory effects of miR-216b and HK2 on cell migration and invasive abilities of BC. The cell migration (Fig. 7B) and invasion (Fig. 7C) were found to be inhibited in the presence of miR-216b mimic (both *p* < 0.05), which was negated by overexpression of *HK2*. These findings demonstrated that miR-216b inhibited the cell proliferation, migration and invasiveness in BC by down-regulating *HK2* in BC.

### MiR-216b promotes BC cell cycle arrest and apoptosis by targeting HK2

**Figure 8:**
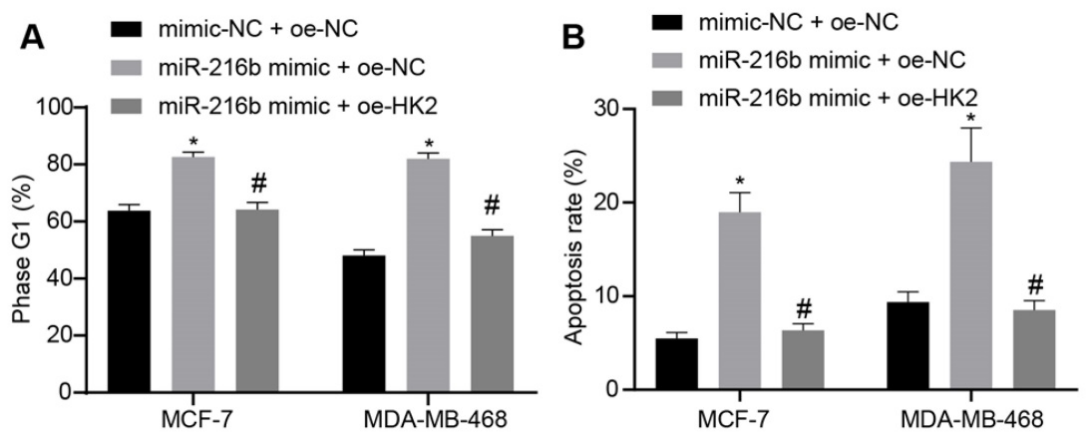
miR-216b promotes cell cycle arrest and apoptosis by targeting *HK2*. A, flow cytometric analysis of cell cycle distribution; B, apoptosis rate after miR-216b upregulation and/or *HK2* overexpression; the experiment was repeated for three times; data were analyzed using one-way ANOVA. *, *p* < 0.05 compared with the cells transfected with mimic NC + oe-NC; #, *p* < 0.05 compared with miR-216b mimic +oe-NC.

Furthermore, the effect of miR-216b and *HK2* on cell cycle distribution and apoptosis of BC cells was evaluated using flow cytometry analyses of PI single staining and Annexin V-FITC/PI double staining. Compared with mimic-NC + oe-NC, treatment with miR-216b mimic + oe-NC significantly blocked the cells at the G1 phase. Meanwhile, miR-216b mimic + oe-HK2 could alleviate the arrest of cells at the G1 phase induced by miR-216b mimic + oe-NC (*p* < 0.05) (Fig. 8A). The results of apoptosis experiment showed that compared with mimic-NC + oe-NC, apoptosis in the miR-216b mimic + oe-NC group was increased; the apoptosis of cells after miR-216b mimic + oe-HK2 treatment could be alleviated by miR-216b mimic + oe-NC (*p* < 0.05) (Fig. 8B). These findings demonstrated that upregulation of miR-216b promoted cell cycle arrest as well as cell apoptosis in BC by targeting *HK2*.

### MiR-216b promotes BC cellular autophagy by targeting HK2

**Figure 9:**
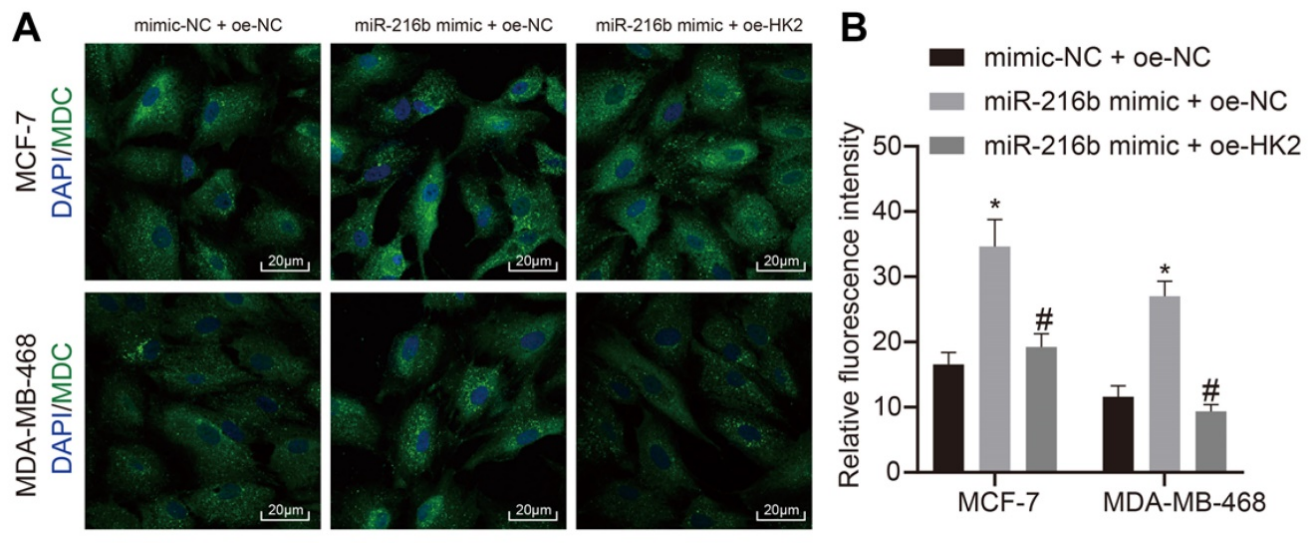
miR-216b promotes cell autophagy by targeting *HK2*. A, representative images of cell autophagy shown by MDC staining (× 500) images; B, statistical data. The images of DAPI staining indicates the nuclei, and merge filed indicates the presence of MDC-stained autolysosomes and DAPI-stained nuclei. B, the intensity of formed autolysosome.*, *p* < 0.05 compared with the cells transfected with mimic NC + oe-NC; #, *p* < 0.05 compared with miR-216b mimic +oe-NC.

In addition, the effects of miR-216b and *HK2* on autophagy of BC cells MCF-7 and MDA-MB-468 cells were analyzed using MDC assay. As demonstrated in Fig. 9A, B, compared with mimic-NC + oe-NC, autophagy was found to be increased in the miR-216b mimic + oe-NC group. The autophagy of cells in the miR-216b mimic + oe-HK2 group could be alleviated by miR-216b mimic + oe-NC (*p* < 0.05). These findings demonstrated that upregulation of miR-216b promotes cell autophagy in BC by targeting *HK2*.

### MiR-216b inhibits tumor growth in vivo

**Figure 10:**
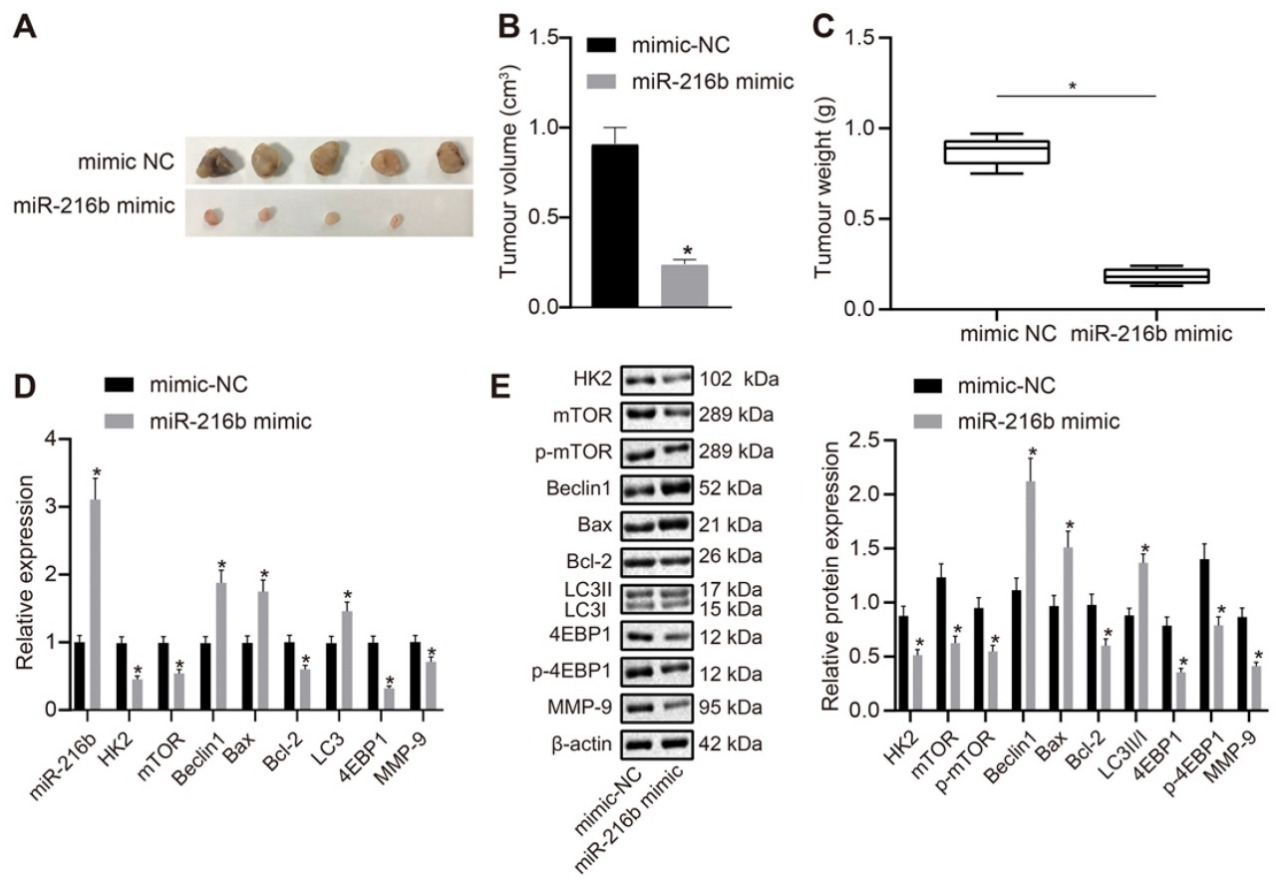
miR-216b inhibits the tumor growth in nude mice. A, representative images of tumors in nude mice after miR-216b upregulation; n = 5 in the mimic-NC group; n = 4 in the miR-216b mimic group; B, tumor volume in nude mice after miR-216b upregulation; C, tumor weight in nude mice after miR-216b upregulation; D, miR-216b expression and mRNA expression of HK2, mTOR, Beclin1, LC3, Bax, Bcl-2, 4EBP1 and MMP-9 after miR-216b upregulation determined by RT-qPCR; E, protein expression of HK2, mTOR, p-mTOR, Beclin1, Bax, Bcl-2, LC3 I, LC3 II, 4EBP1, p-4EBP1 and MMP-9 measured by Western blot analysis; the data were analyzed using unpaired *t* test. *, *p* < 0.05 compared with the nude mice injected with mimic-NC-transfected cells.

Lastly, MDA-MB-468 cells transfected with miR-216b mimic or mimic NC were subcutaneously injected into nude mice so as to explore the effect of miR-216b and *HK2* on *in vivo* oncogenicity of BC cells. The mice were euthanized once the tumors were formed. It was observed that the tumors in the miR-216b mimic group were smaller than those in the NC group (Fig. 10A). Volume and mass of the tumors were also measured, and the results were shown in Fig. 10B-C. The volume and mass of tumors in the miR-216b mimic group were found to be reduced, which suggested that miR-216b inhibited BC progression *in vivo*. The expression of related genes in each group of mice was further detected using RT-qPCR and Western blot (Fig. 10D, E). Compared with the mimic-NC group, the expression of HK2, mTOR, 4EBP1, bcl-2, MMP-9, p-mTOR, and p-4EBP1 was noted to be decreased in the miR-216b mimic group (*p* < 0.05), while that of Beclin1, Bax, LC3 and LC3II/I was increased (*p* < 0.05). Collectively, these findings indicated that upregulation of miR-216b restrained *in vivo* tumor growth of BC.

## Discussion

BC is one of the leading causes of cancer-related death among women worldwide 32. Although 5-year disease-specific survival rates have been improved over the last few decades, a considerable proportion of BC cases are still detected at advanced stages, and show a higher mortality rate due to the advent of drug resistance and increased risk of tumor recurrence 33. Therefore, it is quite necessary to find more effective treatment regimens for BC patients. In lieu of this, the current study set out to uncover the effects of miR-216b on the cellular behavior of BC cells, and obtained findings revealed that miR-216b suppressed BC invasion and migration, and further induced autophagy and apoptosis *via* the mTOR signaling pathway by targeting *HK2*.

Firstly, our findings demonstrated that miR-216b was poorly-expressed in BC. Similarly, Deng *et al.* documented down-regulated expression of miR-216 in nasopharyngeal carcinoma, which were inversely-correlated with tumor metastasis and clinical stage 34. Moreover, the current study unraveled that miR-216b was associated with the progression of BC, as attributed to suppressed BC invasion and migration abilities and induction of autophagy and apoptosis upon over-expression of miR-216b. Meanwhile, a previous study also suggested that lung cancer cells diminished the miR-216b levels to induce the production of autophagy protein Beclin-1 to augment cell survival, which is in accordance with our findings 35. Strikingly, in contrast to other protein kinase RNA-like ER kinase-regulated miRs, miR-216b enhances unfolded protein response-associated apoptosis through regulating a newly identified target, c-Jun 36. These findings and evidence together suggest that miR-216b attenuates the progression of BC.

Interestingly, several studies have highlighted the association of *HK2* with cell proliferation and/or progression in BC 37. Strikingly, immunochemical staining results in our study illustrated that *HK2* was highly expressed in BC tissues. Moreover, we further identified that *HK2* expression was associated with clinical stage, LNM and tumor diameter in BC. As a key glycolytic enzyme for the Warburg effect, HK2 is known to be upregulated in numerous malignancies, and to catalyze the irreversible first step of glucose metabolism 38. In addition, another study has also documented the enrichment of *HK2* in cancer cells, wherein this enrichment resulted in high glycolytic rates in tumors 39. *HK2* is also demonstrated in the metastatic process of pancreatic ductal adenocarcinoma (PDAC) 40. More importantly, findings in our study further demonstrated that miR-216b targets *HK2* and negatively modulates its expression to prevent the progression of BC. Moreover, further experimentation indicated that miR-216b negatively modulate *HK2* expression to suppress cell viability, invasion and migration, as well as to induce autophagy and apoptosis. On the other hand, activation of mTOR signaling pathway has also been previously implicated in astragaloside II (AS II)-induced wound healing 41. Meanwhile, a study has shown that inhibition of the PI3K/Akt/mTOR signaling pathway can positively enhance autophagy in multiple myeloma 42. As a downstream factor of the mTOR signaling pathway, 4EBP1 is known as an indicator of breast tumorigenesis, interplaying with hormone receptor signaling 43. In our study, we observed that miR-216b down-regulates mTOR and 4EBP1, which is indicative of inactivation of the mTOR signaling pathway, which provides evidence for the regulatory role of miR-216b in BC progression.

**Figure 11:**
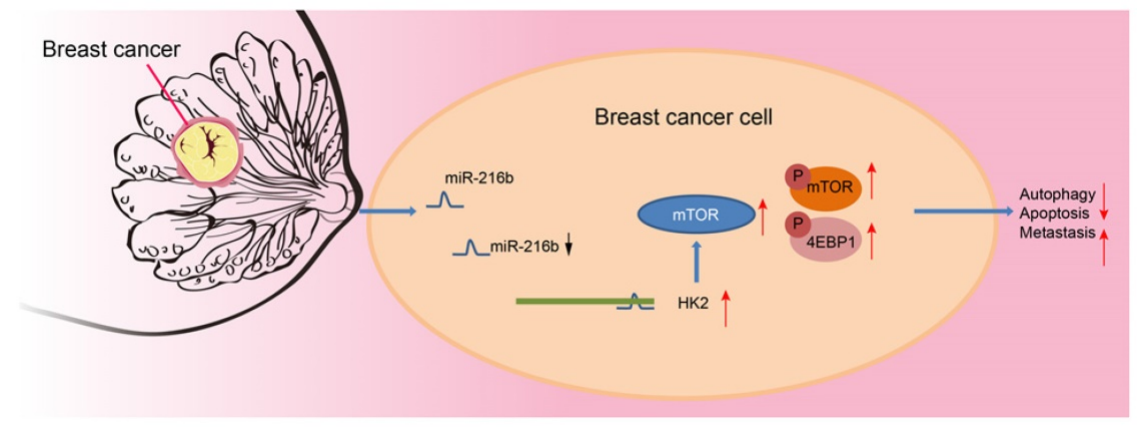
Mechanism. miR-216 down-regulates *HK2* to inactivate the mTOR signaling pathway, thus inhibiting the progression of BC.

Altogether, our results provide strong evidence that upregulation of miR-216b enhances autophagy and apoptosis, as well as suppresses invasion and migration of BC cells *via* blocking the mTOR signaling pathway by targeting *HK2*. Additionally, *in vivo* experimentation in our study demonstrated that transplanted neoplasms were inhibited by miR-216b, which highlights its anti-tumor role (Fig. 11). However, further evidence of the detailed mechanisms of how miR-216b functions with *HK2* and mTOR is warranted to effectively use our findings to improve the life of patients plagued by BC. Nevertheless, we indicate miR-216b as a promising therapeutic target for BC by inhibition of *HK2* and inactivation of the mTOR signaling pathway.

## Patients and Methods

### Ethic statements

All experiments involving humans were performed with the approval of the Ethics Committee of Xinhua Hospital, and signed informed consents were obtained from all participants before the study. All experiments involving animals were conducted in compliance with the Animal Ethics Committee of Xinhua Hospital, and extensive efforts were made to minimize the suffering of the included animals.

### Microarray analysis

Gene expression Profiling Interactive Analysis 2 (GEPIA2) (http://gepia2.cancer-pku.cn) is an updated and enhanced version of GEPIA that supports 198,619 isoforms and 84 cancer subtypes 44. The expression of *HK2* in BC was determined in GEPIA2. Fragments per kilobase million (FPKM) values were transformed into transcripts per kilobase million (TPM) values, which are more comparable between samples. Genotype-tissue expression (GTEx; http://commonfund.nih.gov/GTEx/) provides publicly available gene expression data from 54 normal tissue sites across nearly 1,000 people by RNA sequencing. Normal samples from both TCGA and GTEx (http://commonfund.nih.gov/GTEx/) databases were used for comparisons between cancer and normal tissues.

### Protein-protein interaction (PPI) analysis

Disease genes were retrieved from the DisGeNET database (http://www.disgenet.org/web/DisGeNET/menu) 45, 46. Data of BC were retrieved using the key word “breast carcinoma” in the DisGeNET and the top 10 genes were used for subsequent analysis. PPI analysis was performed using the STRING database (https://string-db.org/).

### Enrichment analysis of Kyoto Encyclopedia of Genes and Genomes (KEGG) pathways

Information including gene function and metabolic pathways was obtained from the KEGG database (http://www.kegg.jp/kegg/). Metabolic pathways related to BC were retrieved using the key word “breast cancer”.

### MiR prediction

miRs possessing the ability to regulate this gene were predicted and obtained from the miRWalk database (http://129.206.7.150/), miRDB database (http://www.mirdb.org/mirdb/policy.html), miR pre-computed target prediction using the RNA22 tool (https://cm.jefferson.edu/rna22/Precomputed/OptionController?species=HomoSapiens&type=mRNA) and microRNA.org database (http://34.236.212.39/microrna/home.do). Subsequently, intersections of predicted miR lists were analyzed with the help of a Venn diagram website (http://bioinformatics.psb.ugent.be/webtools/Venn/).

### Study subjects

BC and adjacent normal tissues were collected from 138 female patients (histopathologically confirmed as BC) at the Xinhua Hospital, School of Medicine, Shanghai Jiao Tong University from November 2016 to September 2018. Patients who received any medicine therapy, chemoradiotherapy or/and immuno-biotherapy before the study were excluded 47. In addition, BC cell lines (MCF-7 and MDA-MB-468), and normal human mammary epithelial cell line (MCF-10A) were purchased from Biobw (Beijing, China). The MCF-7 cells were maintained in minimum essential medium (MEM) supplemented with 10% fetal bovine serum (FBS, 10 μg/mL bovine Insulin and 1% penicillin-streptomycin). MDA-MB-468 cells were cultured in L-15 medium supplemented with 10% FBS. Meanwhile, the MCF-10A cell line was cultured in DMEM-F12 supplemented with 5% horse serum, 20 ng/mL epidermal growth factor (EGF), 100 ng/mL cholera toxin, 10 µg/mL cells were insulin, and 0.5 µg/mL hydrocortisone. All cells were cultured in a 5% CO_2_ incubator at 37°C.

### Immunohistochemistry

Immunohistochemical staining was performed using previously published methods 48. Briefly, BC and adjacent normal tissues were probed with the mouse anti-human monoclonal antibody to HK2 (dilution ratio of 1: 200, ab227198, Abcam, Cambridge, MA) and phosphorylated mTOR (p-mTOR; dilution ratio of 1 : 100, ab109268, Abcam) overnight at 4°C or at 37°C for another 1 h. Meanwhile, biotinylated goat anti-rabbit IgG (ab205718, Abcam) was used as the secondary antibody. Five fields were randomly selected and observed under an optical microscope (Nikon). Judgment criteria for positive-expression were as follows: cell presenting with brown and yellow cytoplasm were regarded to be positively expressed. Five representative high-power fields (positive optical microscope, NIKON, Japan) were also selected for observation and cell counting.

### FISH detection

The paraffin sections were baked in an oven at 60°C for 20 min, and dewaxed for 5 min each in xylene (I, II, III), absolute ethanol (I, II), 90% ethanol (I, II), 80% ethanol (I, II), and 70% ethanol (I, II). The ethanol was washed away in water, and the sections were then transferred to distilled water for 2 min and washed with PBS for 5 min. The sections were boiled in deionized water at 90°C for 15 min. The sections were put in citrate (PH 6.0) and cooked for 40 min, and treated with proteinase K at 37°C for 10 min. The sections were fixed in methanol for 10 min at room temperature, incubated in 70% ethanol, 85% ethanol, and 100% ethanol for 3 min at room temperature, and then dried naturally. The sections were added with pre-hybridization solution and incubated at 42°C for 1 h, After the pre-hybridization solution was aspirated, the sections were added with probe hybridization solution and hybridized overnight at 42°C, and washed twice with buffer for 5 min each time. The sections were naturally dried, and nuclei was stained with 4',6-diamidino-2-phenylindole (DAPI), washed with buffer solution, and then observed and photographed under a microscope for data analysis.

### Cell transfection

The expression patterns of miR-216b in BC cell lines MDA-MB-468 and MCF-7 were determined with the help of reverse transcription quantitative polymerase chain reaction (RT-qPCR), and the cell line exhibiting the highest miR-216b expression levels was selected for subsequent cell experiments. The cells were transfected with plasmids of mimic negative control (NC), miR-216b mimic, inhibitor NC, miR-216b inhibitor, mimic NC + NC over-expression vector (oe-NC), miR-216b mimic + oe-NC, and miR-216b mimic + oe-HK2. miR-216b mimic, miR-216b inhibitor, siRNA against *HK2* (si*HK2*), and siRNA negative control (si-NC) plasmids as shown in Supplementary Table 4 were purchased from Dharmacon Research (Lafayette, CO), with mimic NC, inhibitor NC and siNC as negative controls (NC). Untreated cells as Blank group. MDA-MB-468 and MCF-7 cells (cell density of 3 × 10^5^/well) were seeded in 6-well plates and transfected using lipofectamine 2000 kits (Invitrogen).

### Dual luciferase reporter gene assay

The binding site between miR-216b and the target gene *HK2* was predicted with the help of the microRNA website (www.microRNA.org). A luciferase reporter gene assay was then performed to further verify the binding relationship between miR-216b and *HK2* 49. The p*HK2*-Wt plasmid was constructed with full-length 3'UTR fragment of *HK2* gene inserted into the pmirGLO vector (Promega Corporation, Madison, WI). And p*HK2*-Mut carrier was constructed by PCR-based site-directed mutagenesis. With Renilla luciferase vector pRL-TK (TaKaRa Biotechnology Co., Ltd., Dalian, China) serving as an internal reference, the luciferase activity was measured using a Dual-Luciferase Reporter Assay System (Promega Corporation).

### RT-qPCR

Total RNA content was extracted from the tissues and cells using Trizol kits (Invitrogen). The obtained RNA was reverse transcribed into cDNA using Primescript TMRT reagent kits (RRO37A, TaKaRa Biotechnology Co., Ltd.). The real time PCR was then performed with a fluorescence quantitative PCR instrument (ABI7500, Applied Biosystems, Foster city, CA), with a reaction system consisting of 25 μl of 10 × PCR Buffer, 2.5 μl of 25 m mol/l Mgcl_2_, 1.5 μl of 10 mmol/l dNTP, 0.5 μl of 10 mmol/l Primer, 1 μl of 1 nmol/l Probe, 0.25 μl of Taq, 2.5 μl of cDNA and 15 μl of sterile distilled water. The reaction was repeated in 3 parallel wells. U6 was regarded as the internal reference for miR-216b and GAPDH as the internal reference for other genes (Supplementary Table 5). Relative expression of miR-216b and target genes was calculated using the 2^-ΔΔCt^ method 50.

### Western blot analysis

Total protein content was extracted from the tissues and cells using Protein lysis buffer (C0481, Sigma, St Louis, MO). Following 10% sodium dodecyl sulfate polyacrylamide gel electrophoresis, the proteins were transferred onto a nitrocellulose membrane. The membranes were subsequently incubated with the following antibodies to HK2 (dilution ratio of 1 : 1000, ab104836, Abcam), mTOR (dilution ratio of 1 : 2000, ab2732, Abcam), p-mTOR (dilution ratio of 1:1000, ab109268, Abcam), Beclin1 (dilution ratio of 1 : 500, ab114071, Abcam), Bax (dilution ratio of 1 : 1000, ab77566, Abcam), Bcl-2 (dilution ratio of 1 : 1000, ab692, Abcam), 4EBP1 (dilution ratio of 1 : 1000, ab32130, Abcam), p-4EBP1 (dilution ratio of 1:1000, ab47365, Abcam), LC3A/B (dilution ratio of 1 : 1000, ab128025, Abcam), MMP-9 (dilution ratio of 1 : 1000, ab73734, Abcam) and β-actin (dilution ratio of 1:500, Beijing Cwbiotech Co., Ltd., Beijing, China) overnight at 4°C. Afterwards, the horseradish peroxidase (HRP) -labeled goat anti-mouse or anti-rabbit IgG (SPA131 or SA27, dilution ratio of 1 : 500, Solarbio, Beijing, China) was incubated with the membrane at room temperature for 1.5 h. The relative expression of the proteins was measured as previously described 51.

### Cell count kit-8 (CCK-8)

The transfected MDA-MB-468 and MCF-7 cells (cell density of 2 × 103 cells/mL) were seeded in 96-well plates and incubated with 10 µL of CCK8 reagent (C0037, Beyotime, Shanghai, China) at 37°C for 2 h. Subsequently, the cell viability was detected at 0 h, 24^th^ h, 48^th^, and 72^nd^ h using a microplate Reader (Bio-Rad, Hercules, CA, USA) 52. Cell growth curve was drawn with time point as X-axis and OD value as Y-axis. Three parallel wells were set for each group.

### Scratch test

The transfected cells (cell density of 5 × 10^5^ cells/well) were inoculated in 6 well plates. Scratches were then made using a 20 μL sterile pipette tip. After incubation with serum-free medium, the scratch distance was measured in the cells at 12^th^ h and 24^th^ h under an inverted microscope (Nikon) 53.

### Transwell assay

After 12 h of transfection, the apical Transwell chamber coated with Matrigel (Sigma) was incubated with 200 μL cell suspension, while the lower chamber was added with 500 μL medium containing 100 mL/L FBS (FB-1001/100; Biosera) at 37°C with 5% CO_2_ for 24 h. The invaded cells were observed an inverted microscope (CKX41SF, Olympus, Tokyo, Japan) 51.

### Flow cytometry

After 48 h of transfection, cell apoptosis was analyzed using Annexin-V-FITC cell apoptosis detection kits (C1065, Beyotime). The FITC and PI fluorescence was detected with the help of a BD-Aria flow cytometer (FACSCalibur, Beckman Kurt). The cell cycle distribution was detected after staining with 1% PI containing RNAase for 30 min, on the flow cytometer (FACSCalibur) 54. Three independent experiments were conducted in triplicates.

### Monodansylcadaverine (MDC) staining

MDC, an acid dye, serves as a specific marker for autophagic vacuoles, and thus employed for detection and quantification of autophagy. MDA-MB-468 and MCF-7 cells were seeded in 6-well plates at a concentration of 3 × 10^4^ cells/well in a 5% CO2 incubator at 37°C for 24 h. Following transfection, 50 mM MDC (HY-D1027, MedChemExpress, NJ, USA) was added to each well for 15-min incubation, and then rinsed 3 times with PBS. The nuclei were then stained with DAPI. Finally, the fluorescence was visualized under a confocal laser-scanning microscope (TCS 4D; Leica, Heidelberg, Germany).

### Tumor formation in nude mice

Specific pathogen-free (SPF) nude mice (female, aged 6 weeks, weighing 18-22g) were purchased from Beijing Vital River Laboratories (Beijing, China). The procured animals were housed for 1 week under conditions of suitable temperature and humidity at a 12 h/12 h light/dark cycle. The MDA-MB-468 cells transfected with mimic NC and miR-216b mimic were subsequently harvested. Briefly, the mice were subcutaneously injected with the cell suspension (1 × 10^7^ cells) into the right axilla. The length and width of the xenografts were measured weekly using a vernier caliper, for a total of 5 weeks. The volume (V) was calculated as follows: V (mm^3^) = 1/2 × length × width^2^. At the end of the experiment, the mice were euthanatized and the xenografts were completely excised for further analysis.

### Statistical analysis

Statistical analyses were performed using the SPSS21.0 statistical software (IBM Corp. Armonk, NY). Measurement data were expressed as mean ± standard deviation, and tested for normal distribution and variance homogeneity. Pairwise comparison between BC and adjacent normal tissues were examined using the paired *t* test, while skewed distributed data were tested using non-parametric Wilcoxon rank-sum test. Comparisons among multiple groups were analyzed by one-way analysis of variance (ANOVA). The least significant difference (LSD) test was used for pairwise comparison. When equal variances were not assumed, the nonparametric rank test was applied. Enumeration data were expressed by percentage or ratio, and compared using the chi-square test. Data at different time points were compared using repeated measures ANOVA. The level of statistical significance was set at α = 0.05. A value of *p* < 0.05 was regarded statistically significant.

## Supplementary Material

Supplementary tables 1, 3-5.

Supplementary table 2.

## Abbreviations

BC: breast cancer
DEGs: differentially expressed genes
EMT: epithelial-mesenchymal-transition
GEO: Gene Expression Omnibus
HK2: Hexokinase 2
KEGG: Kyoto Encyclopedia of Genes and Genomes
LSD: least significant difference
MDC: Monodansylcadaverine
mTOR: mammalian target of rapamycin
PDAC: pancreatic ductal adenocarcinoma
PPI: Protein-protein interaction
RT-qPCR: reverse transcription quantitative polymerase chain reaction
