# Supplementary material for: MicroRNA-216b targets HK2 to potentiate autophagy and apoptosis of breast cancer cells via the mTOR signaling pathway
Source: Int J Biol Sci. 2021 Jul 13;17(11):2970–83. doi: 10.7150/ijbs.48933 (PMC8326127; doi:10.7150/ijbs.48933)
Supplement: Supplementary file 1 — Supplementary tables 1, 3-5. [file ijbsv17p2970s1.pdf]

Supplementary Table 1 Top 10 BC-related genes were screened out using DisGeNET database

Gene	Score	PMIDs
PIK3CA	0.280	301
BRCA1	0.280	2228
TP53	0.280	1099
PTEN	0.248	173
AKT1	0.242	152
CHEK2	0.236	130
FGFR2	0.231	112
MDM2	0.224	86
CDH1	0.222	71
TWIST1	0.213	47

Notes: PIK3CA, Phosphatidylinositol-4, 5-bisphosphate 3-kinase catalytic subunit alpha; BRCA1, breast cancer susceptibility gene 1; TP53, tumor protein p53; PTEN, phosphatase and tensin homolog; AKT1, AKT serine/threonine kinase 1; CHEK2, checkpoint kinase 2; FGFR2, fibroblast growth factor receptor 2; MDM2, murine double minute 2; CDH1, E-cadherin; TWIST1, twist family bHLH transcription factor 1; BC, breast cancer； PMID: PubMed Unique Identifier.

Supplementary Table 3 Relationships between miR-216b expression, *HK2* mRNA expression and clinicpathological characters of BC patients

Clinicpathological data	Case	miR-216b expression	*p*	*HK2* mRNA expression	*p*
Age (years)					
< 50	62	0.37 ± 0.04	0.100	1.86 ± 0.21	0.100
≥ 50	76	0.37 ± 0.04		1.86 ± 0.18	
Histological type					
IDC	131	0.37 ± 0.04	0.516	1.86 ± 0.19	0.891
Other type	7	0.38 ± 0.03		1.85 ± 0.12	
Clinical stage					
Stage I/II	85	0.39 ± 0.03	0.0001	1.82 ± 0.19	0.003
Stage III	53	0.33 ± 0.02		1.92 ± 0.18	
LNM					
Yes	58	0.34 ± 0.03	0.0001	1.90 ± 0.19	0.034
No	80	0.39 ± 0.03		1.75 ± 0.16	
Tumor diameter					
≤ 2cm	79	0.40 ± 0.02	0.0001	1.83 ± 0.19	0.030
> 2cm	59	0.33 ± 0.02		1.90 ± 0.18	

Notes: miR-216b, microRNA-216b; *HK2*, Hexokinase-2; BC, breast cancer; IDC, invasive ductal carcinoma; LNM, lymph node metastasis.

Supplementary Table 4 Sequences of mimics, inhibitors and siRNAs

Name	Sequence
miR-216b mimic	Sense: 5’-AAAUCUCUGCAGGCAAAUGUGA-3’<br>Antisense: 5’-ACAUUUGCCUCCAGAGAUUUUU-3’
mimic NC	Sense: 5’-UUCUCCGAACGUGUCACGUTT-3’<br>Antisense: 5’-ACGUGACACGUUCGGAGAATT-3’
miR-216b inhibitor	AAAUCUCUGCAGGCAAAUGUGA
Inhibitor NC	CAGUACUUUUGUGUAGUACAA
siHK2-1	CCGTAACATTCTCATCGATTT
siHK2-2	GCTACAAATCAAAGACAAGAA

Notes: MiR-216b, microRNA-216b; HK2, Hexokinase-2.

Supplementary Table 5 Primer sequences for RT-qPCR

Gene	Sequence
MiR-216b	F: 5′-GCCGCGCTAAAGTGCTTATAGTG-3′
	R: 5′-CACCAGGGTCCGAGGT-3′
HK2	F: 5'-AAGGCTTCAAGGCATCTG-3'
	R: 5'-CCACAGGTCATCATAGTTCC-3'
mTOR	F: 5'-GCTGTACGTTCCTTCTCCTTC-3’
	R: 5'-CAAGAACTCGCTGATCCAAATG-3’
Beclin1	F: *5’-ACCGTGTCACCATCCAGGAA-3’*
	R: *5’-GAAGCTGTTGGCACTTTCTGT-3’*
Bax	F: 5′-CCCGAGAGGTCTTTTTCCGAG-3′
	R: 5′-CCAGCCCATGATGGTTCTGAT-3′
Bcl-2	F: 5′-TACAGGCTGGCTCAGGACTAT-3′
	R: 5′-CGCAACATTTTGTAGCACTCTG-3′
4EBP1	F: 5′ CTAGCCCTACCAGCGATGAG-3′
	R: 5′-CCTGGTATGAGGCCTGAATG-3′
LC3	F: 5′-GCACCATGCCGTCGGAGAAGACC-3′
	R: 5′-CACTCCTAGGTGGGAACACTACTG-3′
MMP-9	F: 5′-ACGCACGACGTCTTCCAGTAC-3′
	R: 5′-ACCTGGTTCAACTCACTCCGG-3′
GAPDH	F: 5'-CATGAGAAGTATGACAACAGCCT-3'
	R: 5'-AGTCCTTCCACGATACCAAAGT-3'
U6	F: 5′-GTGCTCGCTTCGGCAGCACATATAC-3′
	R: 5′-AAAAATATGGAACGCTTCACGAATTTG-3′

Notes: MiR-216b, microRNA-216b; HK2, Hexokinase-2; mTOR, mammalian target of rapamycin; Bcl-2, B-cell lymphoma/leukemia-2; Bax, Bcl-2 Associated X protein; 4EBP1, 4E-binding protein 1; LC3, light chain 3; MMP-9, matrix metalloproteinase-9; GAPDH, glyceraldehyde-3-phosphate dehydrogenase; RT-qPCR, reverse transcription quantitative polymerase chain reaction; F, forward; R, reverse.
